# The Neurobiology of Depression, Burnout and Resilience Among Healthcare Students

**DOI:** 10.1192/bjo.2024.105

**Published:** 2024-08-01

**Authors:** Hamid Alhaj, Abdul Rahman Al Midani, Zainab Alkokhardi, Rashid Abu Helwa, Almuzaffar Al Moukdad

**Affiliations:** University of Sharjah, Sharjah, UAE

## Abstract

**Aims:**

Adapting to academic and social demands may be challenging for university students. Healthcare students are thought to be at high risk of burnout and Major Depressive Disorder (MDD) due to the demands of their training and emotional toll of caring for patients. This risk extends well into physician years, suggesting the persistence of an abnormal psychological state developed during training years. We aimed to investigate the prevalence and severity of depressive symptoms, burnout, and resilience in healthcare students, examine their correlation with salivary cortisol levels, and assess how these factors change during examination periods.

**Methods:**

This longitudinal study investigated the mental health and salivary cortisol levels of medical, dental, or health science students in the UAE at two distinct periods, at the start of the academic semester, and within one week of the examination period. A total of 147 students (51% females) were included, and their demographics and education variables, including cumulative GPA (cGPA), were assessed. Depression, resilience, and burnout scores were measured using the Patient Health Questionnaire-9, Nicholson-McBride Resilience questionnaire, and Maslach Burnout Inventory-Student-Survey, respectively. Participants who met the criteria for MDD were identified. Time-dependent cortisol levels were modelled using functional data analysis and standardised cortisol levels were calculated. Data analysis was done using mixed effect models in R 4.1.2.

**Results:**

Among participants, 12.2% screened positive for MDD at the beginning of the semester, increasing to 16.6% during the examination period. Depression scores were higher during the examination period (p = 0.011). Female gender was significantly associated with higher levels of depression (median difference = 3.00; p < 0.001) and burnout but lower levels of resilience (mean difference = 3.27; p < 0.001). cGPA below 75% (p = 0.009) and history of mental illness (p = 0.015) were associated with increased levels of depression. High cortisol responders (z-value > 1) developed higher depression scores (p = 0.033) compared with low cortisol responders (z-value < −1). Participants with higher resilience were less likely to develop depression and burnout (p < 0.003).

**Conclusion:**

This study shows relatively high levels of depression among healthcare students in the UAE, particularly in females, students with history of mental illness, students with low cGPA, and students with high cortisol levels. Efforts to promote culturally appropriate resilience skills need to be developed to reduce distress and depression in this population.

